# Clinical aspects of *TP53* gene inactivation in diffuse large B-cell lymphoma

**DOI:** 10.1186/s12920-019-0484-9

**Published:** 2019-03-13

**Authors:** Elena N. Voropaeva, Tatyana I. Pospelova, Mikhail I. Voevoda, Vladimir N. Maksimov, Yuriy L. Orlov, Olga B. Seregina

**Affiliations:** 1 Institute of Internal and Preventive Medicine, Branch of Institute of Cytology and Genetics, Siberian Branch of Russian Academy of Sciences, Novosibirsk, Russia; 2 Novosibirsk State Medical University, Novosibirsk, Russia; 3 Novosibirsk State University, Novosibirsk, Russia

**Keywords:** *TP53* gene, Diffuse large B-cell lymphoma, Methylation, Allelic imbalance, Intron mutations, Sequencing

## Abstract

**Background:**

The knowledge about specific mechanisms generating *TP53* dysfunction in diffuse large B-cell lymphoma is limited. The aim of the current study was to comprehensively explore *TP53* gene variability resulting from somatic mutations, promoter methylation, and allelic imbalance in tumorous tissue of diffuse large B-cell lymphoma (DLBCL).

**Methods:**

DNA samples from 74 patients with DLBCL were used. Genomic DNA was isolated from paraffin blocks of lymph nodes or from extranodal biopsies of tumors by the phenol–chloroform extraction method with guanidine. Analysis of coding sequences of the TP53 gene was based on Sanger’s direct sequencing method. The methylation status of the TP53 promoter was analyzed using by methylation-specific PCR on bisulfite-converted DNA. Assessment of the detected mutations was carried out in the IARC *TP53* Database and the *TP53* UMD mutation database of human cancer.

**Results:**

The mutations in regions coding for the DNA-binding domain were prevalent (95%). In the analyzed sample of patients, codons 275, 155, 272, and 212 were hotspots of mutations in the *TP53* gene. In addition, functionally significant intron mutations (IVS6-36G > C and IVS5 + 43G > T) were detected. Instances of *TP53* promoter methylation were observed only in a few samples of diffuse large B-cell lymphoma tissue. Furthermore, loss of heterozygosity was revealed only in the subgroup of patients with altered status of the gene (mutations were detected in five patients and promoter methylation in one case).

**Conclusions:**

Thus, the results suggest that there are two sequential events in the formation of diffuse large B-cell lymphoma in at least some cases. The first event is mutation or methylation of the *TP53* promoter, leading to appearance of a cell with increased risk of malignant transformation. The second event is the loss of an intact allele of the gene; this change is necessary for tumorigenesis. We identified *TP53* mutation patterns in a Russian cohort of patients with de novo DLBCL who were treated with R-CHOP and R-CHOP-like regimens and confirmed that *TP53* mutation status is a valuable prognostic biomarker.

## Introduction

Diffuse large B-cell lymphoma (DLBCL) is characterized by diffuse proliferation of atypical large lymphocytes containing a vesicular nucleus, prominent nucleoli, and basophilic cytoplasm. DLBCL occurs in one third of cases of non-Hodgkin’s lymphoma among adults: up to 25–30% in developed countries and 30–40% in developing countries; these statistics make it one of the most frequent types of lymphoma in the world [1, 2].

An important mechanism underlying the development of DLBCL is the genetic instability of lymphoid cells as part of normal maturation of B cells; this instability can lead to precancerous genetic lesions. As a result, a disturbance of B-cell homeostasis with unregulated proliferation, differentiation blockage, and B-cell immortalization occurs at one of the stages of lymphoid-cell maturation [3].

Genetic factors that disrupt DNA repair or apoptosis may increase the risk of precancerous events. The lesions in the B-lymphocyte genome that had not been repaired or had not been eliminated by apoptosis may be modulated in the future by environmental influences, epigenetic factors (hypo- or hypermethylation), concomitant (autoimmune) diseases, and/or genetic polymorphism and may promote further tumorigenesis [4].

The protein p53 is a nuclear phosphoprotein playing a crucial role in rapid elimination of damaged and potentially dangerous cells [5]. Its tumor-suppressing function results from participation in such processes as cell cycle control, DNA repair, apoptosis, aging, and autophagy through transcription-dependent and -independent mechanisms [6]. Lymphocytes under stress tend to go through p53-dependent apoptosis, in contrast to other cell types, which undergo cell cycle arrest as well as p53-independent apoptosis or necrosis under stressful conditions [7]. For this reason, dysfunction of the *TP53* gene is a basis for initiation and progression of lymphoproliferative disorders [7, 8].

An increase in genetic instability that promotes further tumor progression and allows malignant cells to escape immunosurveillance and therapeutic interventions has been observed in B lymphocytes with an inactivated *TP53* gene. Acceleration of the pace of polyclonal evolution of B cells—with various genetic abnormalities, such as changes in chromosome numbers, chromosomal rearrangements, gene mutations, and amplification of some regions of the genome—takes place under conditions of p53 dysfunction [9].

Dysfunction of the p53 protein may be due to disturbances in the structure of the gene, changes in the transcription process and stability of mRNA or malfunction of post-translational modifications or of interactions of the p53 protein. Probably, molecular mechanisms involving DNA and leading to dysfunction of p53 include gene mutations, promoter methylation, allelic imbalance, and genetic polymorphism [4].

## Materials and methods

The aim of this study was to comprehensively describe the frequency of promoter methylation and that of loss of heterozygosity as well as the frequency, diversity, and functional significance of mutations in coding and intron regions of the *TP53* gene among patients with DLBCL in Novosibirsk, Russia.

### Study population

The study population included 74 patients with DLBCL (35 men and 39 women), aged 21–78 years (52.8 ± 14.3, mean ± SD), who were admitted to Novosibirsk Hematological Center during 2012–2015. As many as 91% of these patients had advanced (III–IV) stages of the disease and two-thirds of them had a poor prognosis according to the International Prognostic Index (IPI). All patients underwent 6–8 cycles of R-CHOP-21 and R-CHOP–like regimens. The Table 1. presents summarizing patients characteristics.

**Table 1:** Clinical features of DLBCL patients at the time of diagnosis (*n* = 74)

	All group (*n* = 74)	*TP53*mut (*n* = 12)	*TP53*wt (*n* = 62)	*P*-value (*TP53*mut vs. *TP53*wt)
Mean age (yrs)	52.8 ± 14.3	50.3 ± 10.6	58.6 ± 18.5	0.347
Sex				0.403
M	35	7	28	
F	39	5	34	
B-symptoms				0.016
No	36	2	34	
Yes	38	10	28	
Performance score				0.352
0 + 1	57	8	49	
2–3	17	4	13	
Stage				0.264
I-II	7	0	7	
III-IV	67	12	55	
Extranodal foci				0.074
No	42	4	38	
Yes	32	8	24	
Splenomegaly				0.044
No	59	7	52	
Yes	15	5	10	
Bone marrow involvement				0.028
No	51	4	47	
Yes	23	8	15	
S-LDH				0.296
Normal	41	5	36	
Elevated	33	7	26	
IPI score				0.018
0–2	21	0	21	
3–5	53	12	41	
Therapy response				
CR	58	7	51	0.066

Abbreviations: *M* male, *F* female, *S-LDH* serum lactate dehydrogenase, *IPI* International Prognostic Index, *CR* complete remission

### Genomic DNA isolation

Genomic DNA was isolated from paraffin blocks of lymph nodes or from extranodal biopsies of tumors by the phenol–chloroform extraction method with guanidine. The tissue sections containing at least 70–80% of tumor cells were chosen for analysis.

### *TP53* gene sequencing and mutation analysis

A prescreening of mutations was not performed. Analysis of coding sequences of the *TP53* gene (from exon 3 to exon 10) and of adjacent intron regions was carried out by Sanger’s direct sequencing method, according to the IARC protocol (2010 update) [10]. At the first stage, single fragments of DNA were produced by PCR, with the genomic DNA as a template. The obtained amplicons were desalted and cleaned up from unincorporated primers and deoxynucleotide triphosphates on microcolumns with Sephadex^ТМ^ G-50 resin (GE Healthcare Bio-Sciences AB).

The sequencing of samples was carried out by the method of capillary electrophoresis on a Hitachi 3500 Genetic Analyzer (Applied Biosystems) with the BigDye® Terminator v.3.1 Kit (Applied Biosystems). Analysis of the sequencing results and alignment and comparison of the obtained data with a reference sequence were conducted in software packages Chromas, SeqScape v.2.7, and Sequence Scanner.

Assessment of biological significance of the detected mutations was carried out in the IARC *TP53* Database and the *TP53* UMD mutation database of human cancer [11, 12].

In addition, bioinformatic analysis of mutations was conducted in SIFT, Mut_ass and on-line program Polymorphism Phenotyping 2 (PolyPhen-2) [13]. To evaluate the biological significance of substitutions in introns, the NetGene2 software was employed [14].

### Methylation-specific PCR

**Fig. 1:**
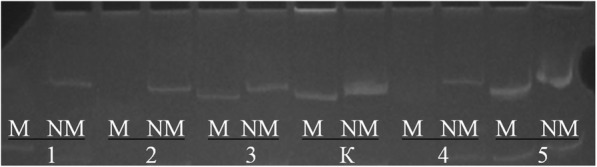
The analysis of promoter methylation status in the *TP53* gene: the results of methylation-specific PCR with flanking primers; electrophoresis in an 8% polyacrylamide gel. M: PCR with primers specific to a methylated allele (length of the product: 166 bp); NМ: PCR with primers specific to an unmethylated allele (length of the product: 170 bp), 1–5: the ID numbers of cases; 1, 2, and 4: normal; 3 and 5: methylation; K: control DNAs

The bisulfite conversion of DNA samples was performed by means of the EZ DNA Methylation Kit (Zymo Research, USA). Three hundred to 500 ng of DNA was used per reaction. Analysis of the methylation status of the *TP53* promoter was carried out by methylation-specific PCR on bisulfite-converted DNA in two microtubes with primers specific to methylated and unmethylated alleles, in accordance with the method described above [15]. For methodological reasons, detection of the methylation status of the *TP53* promoter was performed on tumor samples from 69 patients with DLBCL by the methylation-specific PCR method (Fig. 1).

### Microsatellite analysis

**Fig. 2:**
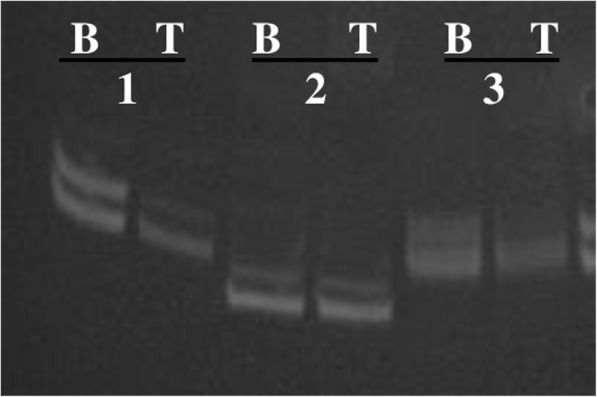
The analysis of microsatellite instability in locus D17S796: the results of PCR with flanking primers, electrophoresis in an 8% polyacrylamide gel (length of the product: 144–174 bp); B: DNA from blood, T: DNA of a tumor tissue, 1–3: the ID numbers of cases; 1, 3: loss of heterozygosity; 2: normal

Assessment of the loss of heterozygosity of *TP53* was carried out at microsatellite locus D17S796 by a PCR method [16]. In this analysis, 24 pairs of samples of normal and tumorous tissue from patients with DLBCL were used (Fig. 2).

### Statistical analysis

A comparison of type frequencies of nucleotide substitutions in *TP53* in DLBCL between the examined sample of patients and data in the IARC *TP53* mutation database was performed by statistical methods: Pearson’s χ^2^ test and Fisher exact test. Clinical and laboratory features were compared using the Fisher exact test. Differences were considered statistically significant at p < 0.05.

## Results

### Mutations in coding and intron sequences of the *TP53* gene

**Fig. 3:**
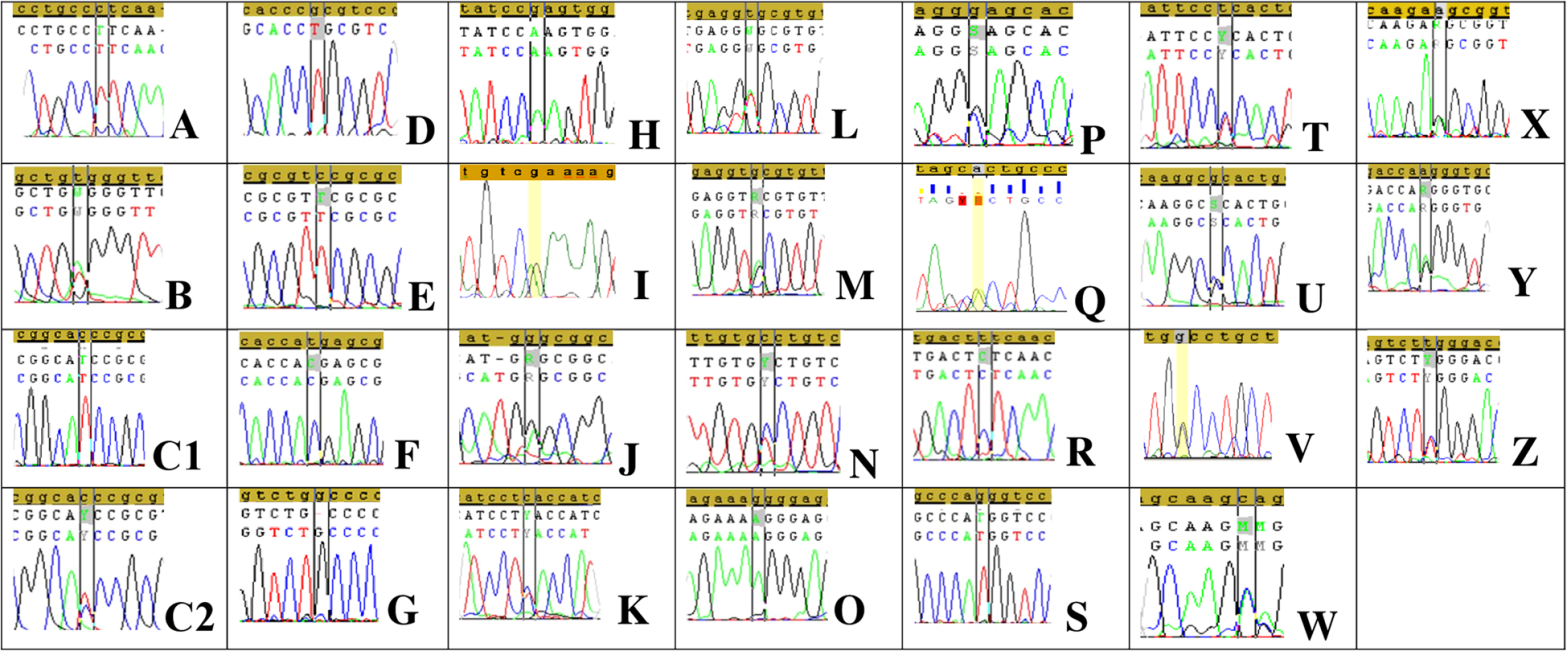
The sequenced fragments of DNA containing mutations of *TP53*. **a**: homozygote of p.L130F, **b**: a heterozygote of p.W146R, **c**1: a homozygote of p.T155I, **c**2: a heterozygote of p.T155I, **d**: a homozygote of p.R156C, **e**: a homozygote of р.V157 V, **f**: a homozygote of р.H179H, **g**: a homozygote of p.A189Pfs, **h**: a homozygote of p.R196Q, **i**: a heterozygote of p.R213Х, **j**: a heterozygote of p.G244S, **k**: a heterozygote of p.L252 L, **l**: a heterozygote of p.V272E, **m**: a heterozygote of р.V272 V, **n**: a heterozygote of p.A276V; **o**: a homozygote of p.G293R, **p**: a heterozygote of p.G302G, **q**: a heterozygote of р.A307A, **r**: a homozygote of IVS4-30Т > С, **s**: a homozygote of IVS5 + 43G > T, **t**: a heterozygote of IVS5-17Т > С, **u**: a heterozygote of IVS6-36G > C, **v**: a heterozygote of IVS7 + 31G > С, **w**: a heterozygote of IVS8 + 10С > А, **x**: a heterozygote of IVS8 + 20A > G, **y**: a heterozygote of IVS8 + 37A > G, and **z**: a heterozygote of IVS9 + 12Т > С

Overall, 33 mutations were revealed: 21 in coding and 12 in intron sequences of *TP53* (Fig. 3). The following distribution of mutations was observed (Table 2): 1 (3%) mutation causing a defect of RNA splicing, 11 (33%) intron mutations with an unknown effect, 12 (37%) missense mutations, 6 (18%) sense mutations, 2 (6%) nonsense mutations, and 1 (3%) frameshift mutation in the *TP53* gene. Except for A189Pfs, all these mutations (96.9%) were single-nucleotide substitutions, 5 (15.6%) of which were mutations of type GC > AT in CpG islands. Substitutions GC > AT constituted 34.4%, GC > CG 3.1%, GC > TA 9.4%, AT>GC 12.5%, AT>CG 12.5%, and AT>TA substitutions represented 12.5%; these results did not significantly differ from the data in the IARC *TP53* mutation database (Fig. 2).

**Table 2:** General characteristics of sequencing results

Intron mutations		In coding sequence of *ТР53* gene			
With unknown effect	Influence on splicing	Nonsense	Frame-shift mutations	Missense	Samesense
IVS4-30Т > С				p.L130F	
IVS5 + 43G > T				p.W146R^a^	
IVS5-17Т > С				p.T155I^a^	р.V157 V
IVS7 + 31G > С^a^				p.R156C	р.H179H
IVS8 + 10С *>* А^a^	IVS6-36G > C	p.R213Х^a^	p.A189Pfs	p.R196Q	p.L252 L
IVS8 + 20A *>* G				p.G244S	р.V272 V
IVS8 + 37A > G				p.V272E^a^	p.G302G
IVS9 + 12Т > С^a^				p.A276V	р.A307A
(rs1800899)				p.G293R	

Note. ^a^Mutations occurring twice in the study population

All mutations in the coding sequences of *TP53* that were identified in our sample of patients with DLBCL had been described earlier in the IARC *TP53* mutations database [11] in other oncological diseases, and these mutations (with the exception of р.A307A) are located in exons 5–8 coding for the DNA-binding domain p53. In the examined sample, 4 (6.8%) patients had multiple mutations, and some findings were revealed repeatedly (each in two cases) (in coding sequences: p.W146R, p.T155I, p.V272E, and p.R213Х; in intron regions: IVS7 + 31G > С, IVS9 + 12Т > С, and IVS8 + 10С > А; see Table 1).

Evaluation of biological significance of all the revealed missense mutations of *TP53* in our patients with DLBCL yielded the following results (Table 3): All three prognostic software tools (PolyРhen-2, SIFT and Mut_ass) classified mutations p.L130F, p.T155I, p.R196Q, p.G244S, p.V272E, and p.A276V (which lead to appearance of a functionally inactive protein) as dangerous, probably pathogenic substitutions, or substitutions with a high/moderate degree of danger. In contrast, mutations р.W146R and p.G293R (slightly decreasing the activity of p53) and mutation p.R156C (hyperactive p53) were regarded as non-pathogenic, neutral substitutions or substitutions with a low/moderate degree of danger.

**Table 3:** The results of functional analysis of missense mutations of *ТР53*

Mutation	Functional prediction			Activity of p53 protein in experiment in vitro [reference]
	PolyРhen-2 (description of mutation)	SIFT (description of mutation)	Mut_ass (degree of pathogenicity)	
p.L130F	Probably pathogenic	Dangerous	High	Not active [44]
p.W146R	Non-pathogenic	Neutral	Low	Slightly reduced [44]
p.T155I	Probably pathogenic	Dangerous	Moderate	Not active [44–46]
p.R156C	Non-pathogenic	Neutral	Low	Hyperactive [37, 44]
p.R196Q	Probably pathogenic	Dangerous	High	Not active [38]
p.G244S	Probably pathogenic	Dangerous	Moderate	Not active [39, 45]
p.V272E	Probably pathogenic	Dangerous	High	Not active [44]
p.A276V	Probably pathogenic	Dangerous	Moderate	Not active [46]
p.G293R	Non-pathogenic	Neutral	Moderate	Slightly reduced [44]
p.A276V	Probably pathogenic	Dangerous	Moderate	Not active [38]
p.G293R	Non-pathogenic	Neutral	Moderate	Slightly reduced [44]

Undoubtedly, mutations p.R213Х and p.A189Pfs have biological significance because each causes emergence of a nonfunctional truncated protein. It is more complicated to evaluate the effect of sense mutations detected in our sample of patients because such mutations are considered synonymous substitutions, i.e., keeping the sense of a codon. According to the prognosis of *TP53* Mutant Assessor, among the sense mutations, substitution р.A307A is noteworthy because codon 307 is near the end of an exon and potentially may be located in a splicing site of an RNA molecule [17].

The functional effects of the majority of intronic mutations revealed in our group of patients with DLBCL are unknown. One of the biologically significant mutations of the *TP53* gene (IVS6-36G > C) is located in intron 6 of the gene. According to the *TP53* UMD mutation database, in human cancer, this mutation means changes that influence splicing [11]. An in vitro experiment indicates that this substitution in the absence of a change in gene coding sequence leads to the survival of cells after chemotherapy and inhibits apoptosis for a long period [18]. According to NetGene2 prognosis, in the group of patients with DLBCL in our study, among the substitutions detected within introns, mutation IVS5 + 43G > Т caused formation of an additional acceptor site of splicing, which is absent under normal conditions. It may lead to inclusion of a part of intron 5 in the sequence of mRNA, premature formation of a stop codon at position 189, and the synthesis of a truncated p53 protein lacking functional activity. Besides, IVS4-30Т > С is outstanding among intron mutations because the alternative gene promoter is placed in intron 4 of *TP53*. This promoter takes part in the synthesis of the delta133 isoform, which is expressed in lymphoid tissue under normal conditions [19].

### Analysis of allelic imbalance and *TP53* methylation status

The frequency of *TP53* promoter methylation in the sample of 69 patients with DLBCL was 4 (5.8%). Promoter methylation frequency did not significantly differ in the subgroups with the mutant and wild-type gene sequence [1 (4.2%) out of 24 vs 3 (6.7%) out of 45, *р* = 0.5663).

Detection of the loss of heterozygosity of *TP53* using microsatellite marker D17S796 was performed on tumor samples from 24 patients with DLBCL, among which 13 patients had mutations, and 11 patients did not have changes in *TP53* sequence. Six (25%) cases of loss of heterozygosity were revealed, among which 5 (83.3%) cases of loss of heterozygosity were detected by the sequencing method in DLBCL tissue samples with mutations in exons 5–8 and adjacent intron regions of the *TP53* gene. Gene promoter methylation was uncovered in one case of the loss of heterozygosity in a DLBCL tissue sample.

### *TP53* mutation status and clinical features of DLBCL

The study cohort was divided into two subgroups: with and without functional *TP53* gene mutations (*TP53*mut and *TP53*wt, accordingly). The *TP53*mut subgroup comprised 12 patients: patients with p.L130F, p.T155I, р.A307A, p.R196Q, p.G244S, IVS6-36G > C and p.A276V mutations, two patients with p.R213Х mutation, two patients with p.V272E mutation and one patient with multiple mutations (p.T155, p.A189Pfs and IVS5 + 43G > T). The clinical features of patient’s subgroups are compared and summarized in Table 1. The analysis demonstrate that *TP53*mut correlated with B-symptoms (*P* = 0.016), splenomegaly (*P* = 0.044) and bone marrow involvement (*P* = 0.028), as well as IPI score of > 2 (*P* = 0.018).

**Fig. 4:**
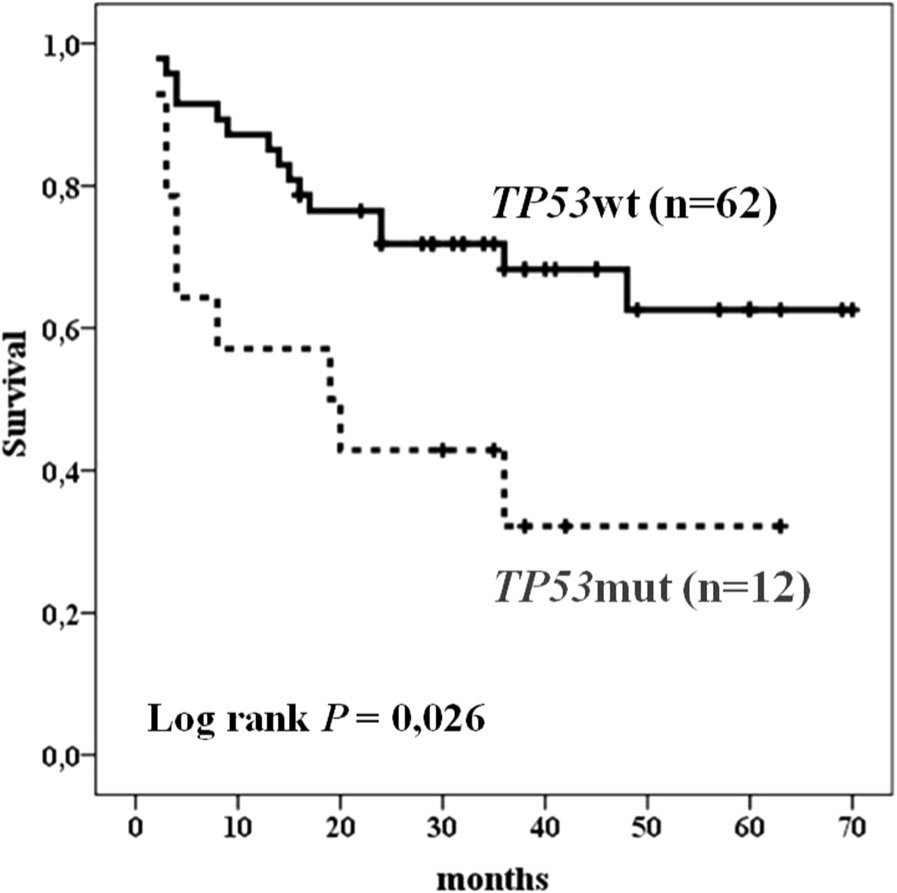
Overall Survival of DLBCL patients with *TP53*mut and *TP53*wt status

DLBCL patients with *TP53*wt tended to had complete remission more often (*P* = 0.066, Table 1) and had better overall survival (OS) (*P* = 0.026, Fig. 4) compared with DLBCL patients with *TP53*mut. The 5-year OS was 69.4% for patients with *TP53*wt versus 41.7% for those with *TP53*mut DLBCL. The median OS of DLBCL patients with *TP53*mut was 20 months. In contrast, median OS of DLBCL patients with *TP53*wt was not achieved. Univariate analysis showed, that extranodal foci, IPI score of > 2 and *TP53* mutations predicted decrease OS of DLBCL patients. Multivariate analysis showed that IPI score were the only prognostic factors that independently predicted worse OS of DLBCL patients treated with R-CHOP and R-CHOP-like regiments. Patients with IPI score of > 2 had a three times hazard for OS (*P* = 0.005) compared with patients with IPI score of ≤2 (Table 4).

**Table 4:** Univariate and multivariate analysis of OS predictors of patients with DLBCL

	HR	95% CI	*P*-value
Univariate analysis			
B-symptoms	2.401	0.530–0.876	0.256
Performance score 2–3	1.938	0.905–4.149	0.088
Stage III-IV	2.025	0.576–7.116	0.271
Extranodal foci	3.233	1.140–9.951	0.029
Bone marrow involvement	1.539	0.501–4.722	0.451
Elevated S-LDH	1.073	0.900–9.438	0.068
IPI score > 2	2.844	1.384–5.842	0.004
*TP53*mut	2.707	1.077–6.800	0.034
Multivariate analysis			
IPI score > 2	2.994	1.405–6.381	0.005
*TP53*mut	2.128	0.627–7.217	0.226
Extranodal foci	2.405	0.787–7.369	0.125

Abbreviations: *S-LDH* serum lactate dehydrogenase, *IPI* International Prognostic Index, *HR* hazard ratio, *CI* confidence interval

Because of the small cohort of DLBCL patients with other *TP53* aberrations in our study, we do not present the analysis of prognostic and predictive impact of loss of heterozygosity and methylation.

## Discussion

Analysis of literature data has shown that deletion of 17p13.1 leading to the loss of heterozygosity of *TP53* has been registered at different frequencies in various studies: from 30.4 to 42% according to Chinese authors [20–22], from 40 to 50.4% in the Czech population [23, 24], 30.4% in the Arab population [25], and 22.5% in the Austrian population [26]. The lowest frequency of 17p13.1 deletion (22.2%) is reported in the International DLBCL Rituximab-CHOP Consortium Program Study [27], combining the samples of patients from 16 hematological centers in the USA, Switzerland, Holland, Germany, Italy, and Spain.

The most actively studied topic on *TP53* gene variability in DLBCL is the analysis of its coding sequences revealing the presence of mutations. It has been shown that mutation frequency in the *TP53* gene in DLBCL is 20% or higher [28].

In 1990, the IARC *TP53* mutation database was created for documenting the mutations in this gene and contains information on more than 30,000 somatic and 700 germ-line mutations at present [24].Research in this database has revealed that in DLBCL, more than 120 mutations of *TP53* have been described; 95% of them are single-nucleotide substitutions: 88% are missense mutations, 7% are nonsense mutations, and 5% are frame-shift mutations. More than 95% of mutations have been detected in exons 5–8, but mutations in exons 9–11 have not been described.

The following codons are hotspots of mutations in *TP53* in relation to DLBCL (in the order of decreasing frequency): 248, 273, 175, 245, 281, 244, 305, 249, and 297 (http://p53.iarc.fr/DownloadDataset.aspx). Nonetheless, the prevalence of so-called hotspot mutations may change depending on the type of cancer and the ethnic origin of patients [29]. Comparative analysis of *TP53* mutations indicates that their diversity and frequencies may significantly vary too, depending on the population under study [30]. There is no information about a Russian population in the current version of the IARC *TP53* mutation database.

The vast majority of studies on the role of changes in the nucleotide sequence of *TP53* have focused on exons 5–8. The intron regions have hardly been researched. Nevertheless, these sequences may potentially influence not only splicing of RNA but also gene expression by disturbing the processing autoregulation, normal post-transcriptional mRNA modifications, and post-translational protein modifications [18, 31, 32].

The relation between hypermethylation of the *TP53* promoter and downregulation of gene transcription has been revealed in some tumors [4, 33, 34]. Despite thorough exploration of this gene’s methylation in cancer, this topic has been insufficiently studied in hematological cancers and hardly addressed in lymphoproliferative diseases [4]. For example, methylation of the *TP53* promoter is observed in one-third of patients with acute lymphoblastic leukemia [35] and in one-fifth of patients with chronic lymphocytic leukemia [4]. There is only anecdotal evidence on the frequency of methylation of the *TP53* promoter in DLBCL [36]. Comprehensive characterization of *TP53* gene variability in DLBCL has not been carried out yet.

The aim of this study was to comprehensively describe the frequency of promoter methylation and that of loss of heterozygosity as well as the frequency, diversity, and functional significance of mutations in coding and intron regions of the *TP53* gene among patients with DLBCL in Novosibirsk, Russia.

The diversity of single-nucleotide substitutions detected in tumor samples from our group of patients with DLBCL did not significantly differ from that in the IARC *TP53* mutation database. Mutations in regions encoding the DNA-binding site were predominant (95%). Mutation p.G293R is the only revealed missense substitution that does not affect the functionally significant DNA-binding domain of p53.

Searches in the IARC *TP53* mutation database showed that all the functionally significant mutations detected in our study population had been described previously in a wide range of human cancers. Codons 196 and 213 in various cancers and codon 244 in hematological cancers in general and in DLBCL in particular are hotspots of mutations in *TP53* (http://p53.iarc.fr/DownloadDataset.aspx).

Moreover, Li-Fraumeni syndrome (http://p53.iarc.fr/DownloadDataset.aspx) has been described, which is characterized by the development of cancer in different parts of the body because of a germline mutation, p.R213Х, р.G244S, p.L130F, or p.T155, similar to the mutations described in our study.

In this study, mutations were not detected in the majority of codons (248, 273, 175, 245, 281, 305, 249, and 297, except codon 244) for which most of the mutations in *TP53* have been described in DLBCL in the IARC *TP53* mutation database. Codons 275, 155, 272, and 212 were found to be hotspots of mutations in the analyzed sample of patients.

Review of the published literature [37–40] about the consequences of mutations in the *TP53* gene showed that each of these mutations may have multidirectional effects on different functions of p53. These effects may be tentatively subdivided into the consequences for protein structure, its biochemical properties, and biological activity and, in some cases, lead to the emergence of new functions of p53, absent in the wild-type protein.

Thus, each of the mutant forms of the protein is a unique product of a mutation and may combine an increase and/or decrease in a particular type of p53 activity and may alter its structure or generate new properties.

The emergence of a functionally inactive protein p53 in the analyzed group of patients with DLBCL was caused by one of missense mutations—p.L130F, p.T155I, p.R196Q, p.G244S, p.V272E, or p.A276V—together with mutation p.A189Pfs, leading to frameshift mutations, nonsense substitution p.R213Х, or splicing mutation IVS6-36G > C.

All the above events in the coding part of the gene—that affect the sequence carrying information about highly conserved sites of the DNA-binding domain of p53—were fixed during evolution (in phylogeny) and occur in most isoforms of p53 and in the structure of homologous proteins p63 and p73.

Mutations p.R213Х and р.G244S as well as p.V272E have already been described in DLBCL [41, 42], and р.T155I (detected in our study) has been previously detected in tumor samples from some patients with chronic lymphocytic leukemia and is known to be associated with poor prognosis and a weak response to treatment [43].

Among cases of hematological cancers, mutation p.V272E has been described in Burkitt’s lymphoma, Hodgkin’s lymphoma, and B-cell non-Hodgkin’s lymphoma (http://p53.iarc.fr/DownloadDataset.aspx).

All these findings are suggestive of selection of p.L130F, p.T155I, p.R196Q, p.G244S, p.V272E, p.A276V, p.R213Х, and p.A189Pfs at various stages of cancer progression [37–39, 44–46]; therefore, their detection in tumor samples from patients with DLBCL is not coincidental.

Our findings indicate that only two missense substitutions (p.W146R and p.G293R)—among all the revealed cases in the analyzed group of patients with DLBCL—did not significantly influence the function of p53. In contrast, p.R156C led to the appearance of a hyperactive mutant protein.

Out of all the mutations detected in our study, only two may influence splicing of RNA. These include samesense substitutions р.A307A and IVS6-36G > C. The functional significance of IVS6-36G > C has been proved in an in vitro experiment [18]. According to the prediction of *TP53* Mutant assessor (release 1.00, 2012), р.A307A is also located at a splicing site of RNA [17]. Even though in sense mutations, the new codon continues encoding the same amino acid, it is believed that this type of mutations may change splicing, transcription, and/or stability of RNA [11].

The functional effects of most intron and sense mutations detected in our group of patients with DLBCL (but not discussed) remain unknown. It is likely that intron mutations may influence not only RNA splicing but also gene expression control, by causing gain- or loss-of-function regulatory elements in *TP53* region, thereby creating or disrupting binding sites for certain DNA-sequence-specific transcription factors that interfere with normal activation or autoregulation of *TP53*, and possibly transcriptional regulation of other potential downstream genes [31, 47]. Furthermore, intron mutation IVS4-30Т > С is noteworthy because an alternative gene promoter is placed in intron 4 of *TP53* and takes part in the synthesis of delta133 and delta160 isoforms of p53 [19]. Thus, whether these newly identified intron mutations of *TP53* are functional warrants further investigation.

Some published reports about low frequencies of *TP53* promoter methylation in DLBCL were verified in our group of patients. The frequency of *TP53* promoter methylation in the analyzed group of patients with DLBCL was 5.8% and did not differ significantly from subgroups with mutant (4.2%) and wild-type (6.7%) gene structure or from the findings of K. Amara and coauthors (3.7%) [36].

Analysis of the loss of heterozygosity of *TP53* was performed on 24 tumor samples of DLBCL in our study population, and 13 of them were found to have mutations, whereas 11 did not have changes in the sequences of *TP53*. Our investigation of microsatellite marker D17S796, which is located near *TP53*, detected 6 (25%) cases of loss of heterozygosity, in agreement with the literature data [27].

Furthermore, loss of heterozygosity was observed only in the subgroup of patients with a alteration of the *TP53* gene (mutations were revealed in 5 tumor samples, and promoter methylation in one tumor), which accounted for 6 (42.9%) out of 14 versus 0% in the group of 10 patients with the intact gene (*р* = 0.0223).

Our comprehensive assessment of gene variability indicates that the dysfunction of p53 in DLBCL may emerge via a two-hit mechanism. According to this model, two sequential events are necessary for transformation of a normal B cell into a cancerous cell during carcinogenesis of at least some cases of DLBCL. The first event is a mutation or methylation in the *TP53* promoter, giving rise to a cell with increased risk of malignant transformation. The second event is the loss of an intact allele of *TP53* in the cell; this change is necessary for tumorigenesis.

Thus, in DLBCL, our results proved the selection of functionally significant mutations in the gene regions encoding the DNA-binding domain of p53.

In the analyzed sample of patients with DLBCL, the location of hotspots of mutations (in contrast to the set of single-nucleotide substitutions) is different from the data listed in the IARC *TP53* Mutation database.

The presence of important intronic and samesense substitutions was demonstrated and confirmed the importance of studying the noncoding gene regions adjacent to exons and of bioinformatic analysis of the uncovered synonymous substitutions. In total, our results show that *TP53* mutation status is a prognostic factor that stratifies DLBCL patients treated with R-CHOP and R-CHOP-like regimens. This observation is a further supports the crucial role of p53 in death of tumor cells and tumor suppression. In Russian cohort of de novo DLBCL patients, we show that *TP53*mut are correlated with B-symptoms, splenomegaly and bone marrow involvement, as well as adverse IPI prognostic groups. Nonetheless, DLBCL patients with *TP53*wt tended to had complete remission more often (*P* = 0.066).

It is known, that despite the addition of rituximab to therapy, *TP53* mutation is an independent prognostic factor that predicts poor survival in patients with DLBCL [28]. To eliminate the possible impact of *TP53*mut on OS, we also performed survival analysis for patients study cohort and found that patients with *TP53*wt had significantly better OS (*P* = 0.026). These data are in agreement with a recent study [27].

Our results show that together with extranodal foci and IPI score of > 2 *TP53*mut predicted decrease OS of DLBCL patients. In fact, *TP53* mutation status is an independent prognostic factor in patients with DLBCL treated with R-CHOP [28]. But multivariate analysis show that the impact on survival DLBCL patients of IPI score of > 2 is more pronounced than the impact of *TP53*mut. This might be because of the small cohort of DLBCL patients with *TP53*mut in our study.

## Conclusions

In conclusion, in the present study, we identified *TP53* mutation patterns in a Russian cohort of patients with de novo DLBCL who were treated with R-CHOP and R-CHOP-like regimens and confirmed that *TP53* mutation status is a valuable prognostic biomarker. Therefore, therapeutic approaches targeting the inactivated *TP53* pathway may further improve clinical outcomes of patients with DLBCL.

Because of the small cohort of DLBCL patients with other *TP53* aberrations in our study we do not present the analysis of prognostic and predictive impact of LOH and methylation. However, the comprehensive analysis of *TP53* status gives us better insights into the possible mechanisms behind participation of this gene’s variability in the pathogenesis of DLBCL. It was shown here that dysfunction of p53 in DLBCL may emerge according to the two-hit principle.

## Abbreviations

CI: Confidence Interval
CR: Complete Remission
DLBCL: Diffuse Large B-cell Lymphoma
HR: Hazard Ratio
IARC: International Agency for Research on Cancer
IPI: International Prognostic Index
OS: Overall Survival
R-CHOP: Rituximab, Cyclophosphamide, Hydroxydaunorubicin, Oncovin, Prednisone
SIFT: Sorting Intolerant From Tolerant
S-LDH: Serum Lactate Dehydrogenase
